# Supplementary material for: Increasing large wildfires over the western United States linked to diminishing sea ice in the Arctic
Source: Nat Commun. 2021 Oct 26;12:6048. doi: 10.1038/s41467-021-26232-9 (PMC8548308; doi:10.1038/s41467-021-26232-9)
Supplement: Supplementary file 1 — Supplementary Information [file 41467_2021_26232_MOESM1_ESM.pdf]

Title: Increasing Large Wildfires over the Western United States Linked to Diminishing Sea Ice in the Arctic

Authors: Yufei Zou[1*†], Philip J. Rasch[1], Hailong Wang[1*], Zuowei Xie[2] & Rudong Zhang[1]

[1]Atmospheric Sciences and Global Change Division, Pacific Northwest National Laboratory, Richland, WA 99354, USA.

[2]International Center for Climate and Environment Sciences, Institute of Atmospheric Physics, Chinese Academy of Sciences, Beijing, 100029, China.

*Email: yufei.zou@pnnl.gov; hailong.wang@pnnl.gov

[†]Now at: Our Kettle, Inc., Kensington, CA 94707, USA.

Supplementary Information:

Supplementary Discussion

Supplementary Figures 1-17

Supplementary Tables 1-5

Supplementary References (1-28)

Supplementary Discussion:

Robust teleconnection in different fire weather and reanalysis datasets. There are multiple fire weather indices (e.g., FFWI[1]; FWI[2]) derived from different reanalysis datasets available for analyzing the teleconnection relationship between Arctic sea-ice loss and regional fire weather changes. We choose to use FFWI rather than FWI in the main text to keep consistency between reanalysis-based and model-based analyses because FFWI does not require high-frequency hourly meteorological input data that are not available from most modeling outputs for its calculation. Since FFWI is based on surface air temperature, 2-m relative humidity, and surface wind speed without precipitation, we used both FFWI and precipitation (e.g., Fig. 4 in the main text; Supplementary Figs. 3/8) to get a more comprehensive view of surface fire weather conditions. Here we also use two FWI datasets[3,4] derived from different reanalysis data (i.e., ERA5[5]; MERRA-2[6]) to cross-validate the identified teleconnection between the Arctic sea ice and regional fire weather changes as described by ERA5-based FFWI in the main text.

The FWI datasets used for cross-validation purpose are derived from the ERA5 reanalysis data (*T*, RH, precipitation, and wind speed) by Vitolo et al.[3] and the MERRA-2 reanalysis data (T, RH, wind speed, snow depth, and bias-corrected precipitation) in the Global Fire WEather Database[4] (GFWED; https://data.giss.nasa.gov/impacts/gfwed/), respectively. We first calculate the correlations between each pair of the three fire weather index datasets (i.e., ERA5-based FFWI; ERA5-based FWI; MERRA-2-based FWI) as well as their correlations with the HadISST-based SIC time series (Supplementary Table 1). The correlation coefficients among three pairs of the fire weather indices range from 0.71 (between ERA5-based FFWI and MERRA-2-based FWI) to 0.95 (between ERA5-based FWI and MERRA-2-based FWI), with all correlations statistically significant at the 0.001 level. Moreover, the time series of all three fire

weather index data show statistically significant correlations with the SIC time series with the lowest correlation between MERRA-2-based FWI and SIC (r=-0.37; p-value=0.02) and the highest correlation between ERA5-based FFWI and SIC (r=-0.68; p-value<0.001).

We further re-examine the changes in spatial distributions of regional fire weather as described by the MERRA-2-based FWI data between the SIC- and SIC+ years like what we show by Fig. 1 in the main text. We believe this is a conservative evaluation because of the lowest correlation (but still statistically significant at the 0.05 level) between MERRA-2-based FWI and SIC. As shown by the spatial pattern in Supplementary Fig. 2a, most of the western U.S., especially the Southwestern U.S., also show largely increased FWI values during the SIC- years than the SIC+ years. This is generally consistent with the result shown by the ERA5-based FFWI data in Fig. 1a, albeit the regions with statistically significant fire weather changes expand northward in the ERA5-based FFWI result. Similarly, the months showing significant regional fire weather changes are fewer as suggested by MERRA-2-based FWI (Supplementary Fig. 2c) than that by ERA5-based FFWI, but both data agree that more fire-favorable weather with elevated fire weather indices would occur in autumn and early winter following sea-ice loss in preceding summer. All these results of consistent spatial and temporal changes in regional fire weather confirm a robust linkage between decreasing Arctic sea ice and worsening regional fire weather as described by different fire weather indices (FFWI and FWI) derived from two reanalysis datasets (ERA5 and MERRA-2).

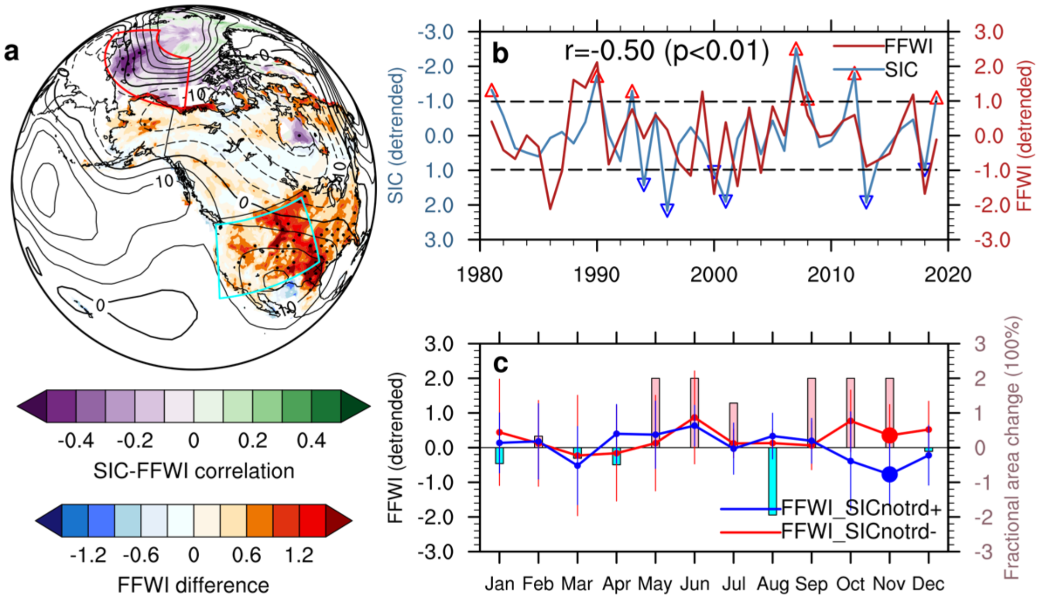

Supplementary Fig. 1. Observation- and ERA5 reanalysis-based Arctic sea ice and regional fire teleconnection after removing the long-term trends. a, spatial distributions of the correlation (shading in the Arctic denoted by the purple-green color bar) between seasonal average detrended Arctic sea-ice concentrations in summer and autumn (July to October) and seasonal and regional average detrended FFWI over the western U.S. in the following autumn and early winter (September to December), and the difference of seasonal average detrended FFWI (shading in North America denoted by the blue-red color bar) between years with minimum (SICnotrd-: red up-pointing triangles in **b**) and maximum (SICnotrd+: blue down-pointing triangles in **b**) detrended Arctic SIC. The difference of seasonal (September to December) average detrended geopotential height at 500 hPa between the SICnotrd- and SICnotrd+ years is also shown (contours with negative values in dashed lines; unit: m). Stipples in **a** mark regions that are significantly different from 0 at the 0.05 significance level of two-sided t-tests. **b**, time series of regional and seasonal average detrended SIC (seasonal mean from July to October; first normalized by its 1981-2010 climatological mean and standard deviation

and then detrended; note its scale on the left Y-axis is inverted to directly compare temporal variations of both time series), detrended FFWI (seasonal mean from September to December), and their correlation. The region definitions for the Pacific sector of the Arctic and the western U.S. are outlined by the red and cyan boxes in **a**, respectively. The horizontal dashed lines denote the ±1 standard deviations of normalized and detrended SIC as thresholds for selecting the SICnotrd+/- years. **c**, the composite of monthly detrended FFWI (solid lines with dots and error bars) and fractional detrended burned area change of large wildfires (vertical bars) over the western U.S. Error bars in **c** denote ±1 standard deviations of monthly detrended FFWI in each group. The larger dot for monthly FFWI in **c** denotes the 0.05 significance level of two-sided t-tests for monthly FFWI differences between years with minimum (FFWI_SICnotrd-) and maximum (FFWI_SICnotrd+) detrended Arctic SIC.

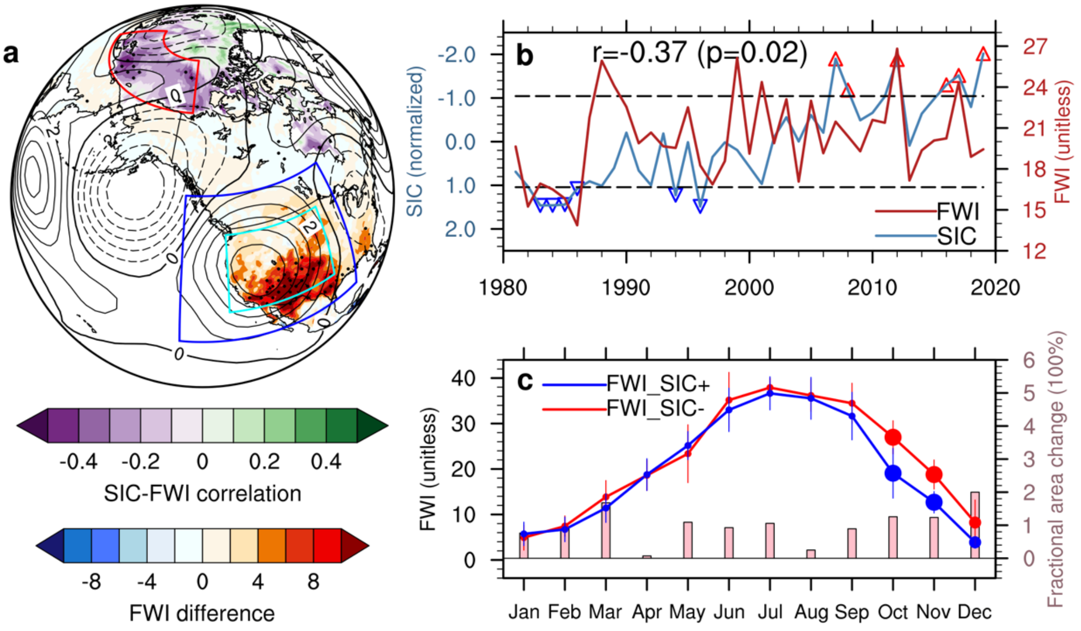

Supplementary Fig. 2. Observation- and MERRA-2 reanalysis-based Arctic sea ice and regional fire teleconnection. a, spatial distributions of the correlation (shading in the Arctic denoted by the purple-green color bar) between seasonal average Arctic sea-ice concentrations in summer and autumn (July to October) and seasonal and regional average FWI over the western U.S. in the following autumn and early winter (September to December), and the difference of seasonal average FWI (shading in North America denoted by the blue-red color bar) between years with minimum (red up-pointing triangles in **b**) and maximum (blue down-pointing triangles in **b**) Arctic SIC. The regression of geopotential height anomalies at 500 hPa onto the seasonal and regional average FFWI is also shown (contours with negative values in dashed lines; unit: m). Stipples in **a** mark regions that are significantly different from 0 at the 0.05 significance level of two-sided t-tests. **b**, time series of seasonal and regional average SIC (seasonal mean from July to October; normalized by its 1981-2010 climatological mean and standard deviation; note its scale on the left Y-axis is inverted to directly compare temporal variations of both time series) and FWI (seasonal mean from September to December) and their

correlation. The region definitions for the Pacific sector of the Arctic, the western U.S., and the Z500i projection region are outlined by the red, cyan, and blue boxes, respectively, in **a**. **c**, the composite of monthly FWI (solid lines with dots and error bars) and fractional burned area change of large wildfires (vertical bars) over the western U.S. Error bars in **c** denote ±1 standard deviations of monthly FWI in each group. Dot sizes for monthly FWI in **c** denote the 0.05 (large), 0.1 (medium), and non-significant (small) significance levels of two-sided t-tests for monthly FWI differences between years with minimum (FWI_SIC-) and maximum (FWI_SIC+) Arctic SIC, respectively.

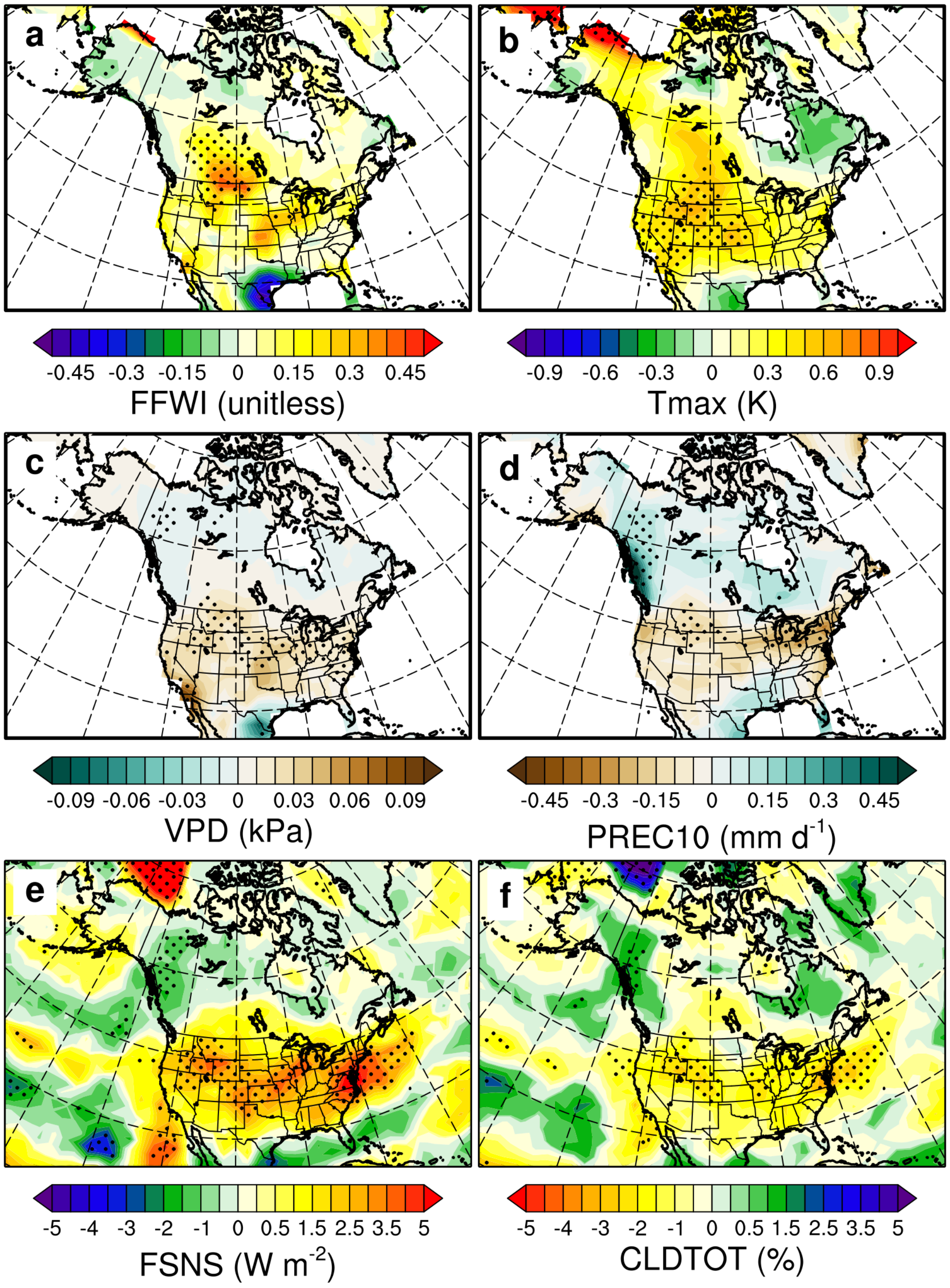

Supplementary Fig. 3. Fire-related weather responses in the CESM-RESFire experiments. **a**, the difference of seasonal average (September to December) FFWI between the SICexp- and SICexp+ sensitivity experiments. **b**, as in **a**, but for the difference of seasonal average daily maximum of 2-m temperature (Tmax). **c**, as in **a**, but for the difference of seasonal average

surface vapor pressure deficit (VPD). **d**, as in **a**, but for the difference of seasonal average 10-day running mean of total precipitation (PREC10). **e**, as in **a**, but for the difference of seasonal average net solar flux at surface (FSNS; positive for the downward direction). **f**, as in **a**, but for the difference of seasonal average vertically integrated total cloud fraction (CLDTOT). Stipples in **a-f** show regions that are significantly different from 0 at the 0.1 significance level of a two-sided t-test.

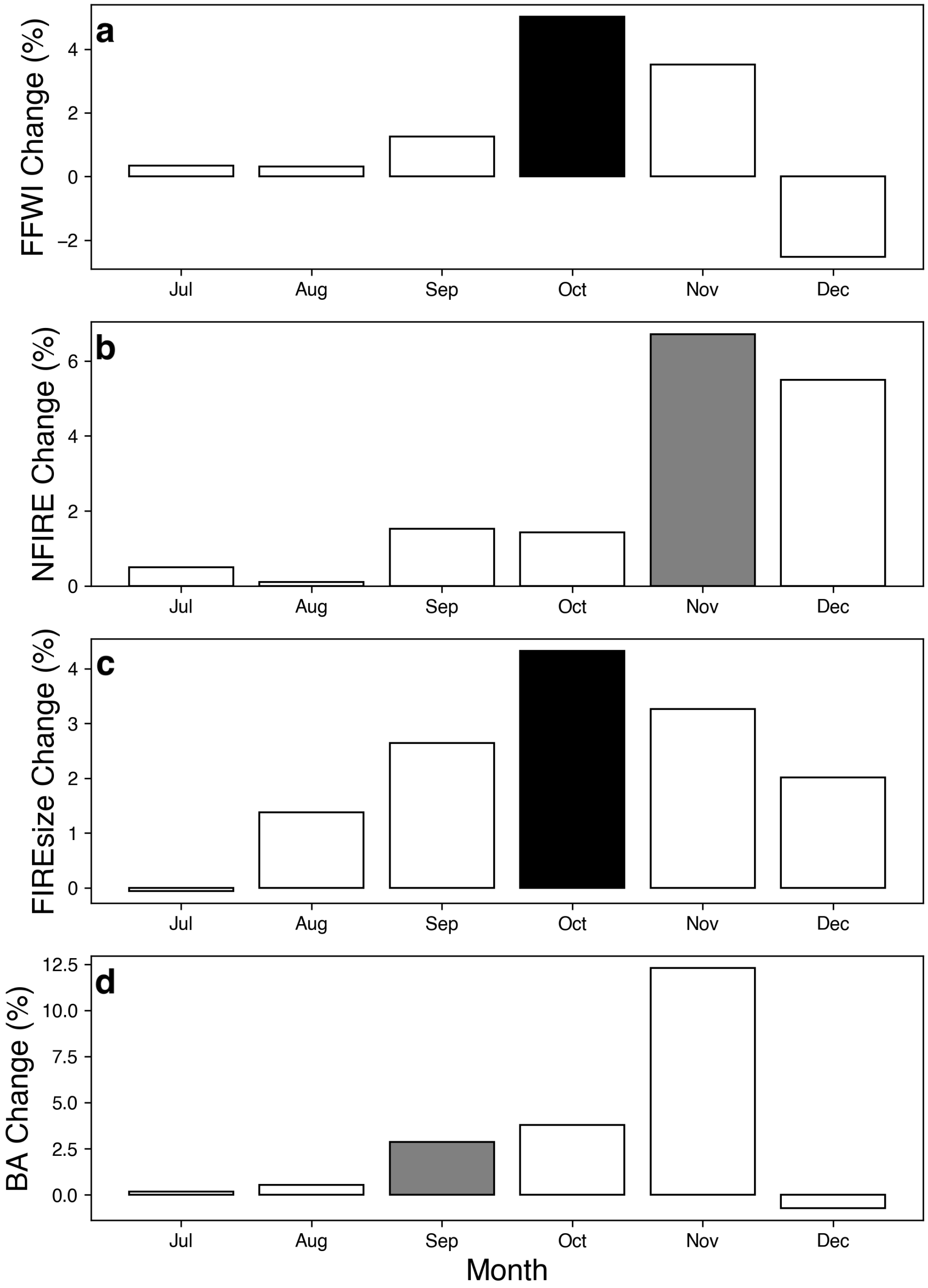

Supplementary Fig. 4. Fractional changes of monthly fire weather and fire variables in the CESM-RESFire experiments. a, fractional Changes of regional average FFWI over the western U.S. between the SICexp- and SICexp+ sensitivity experiments. **b**, as in **a**, but for fractional changes of regional total fire counts. **c**, as in **a**, but for fractional changes of regional average fire

sizes. **d**, as in **a**, but for fractional changes of regional total burned area. Shadings in **a-d** show fractional changes that are significantly different from 0 at the 0.05 (dark) and 0.1 (grey) significance levels of a two-sided t-test.

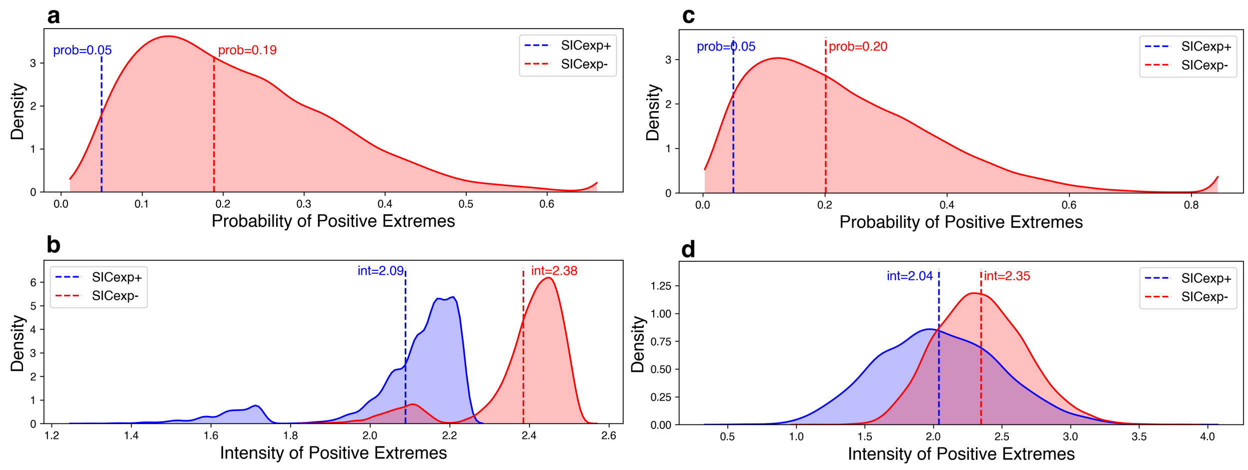

Supplementary Fig. 5. Probability and intensity changes of extreme burning years in the CESM-RESFire experiments. a, the statistical distribution for occurrence probability estimates of extreme burning years in the SICexp- and SICexp+ climate sensitivity experiments based on the bootstrap resampling method without replacement (see Methods). The vertical dashed lines denote ensemble mean probabilities for the SICexp- (red) and SICexp+ (blue) experiments. **b**, as in **a**, but for intensity estimates of extreme burning years without replacement in the bootstrap estimation. The vertical dashed lines denote ensemble mean intensity for the SICexp- (red) and SICexp+ (blue) experiments. **c-d**, as in **a-b**, but for the probability and intensity estimates of extreme burning years with replacement in the bootstrap estimation.

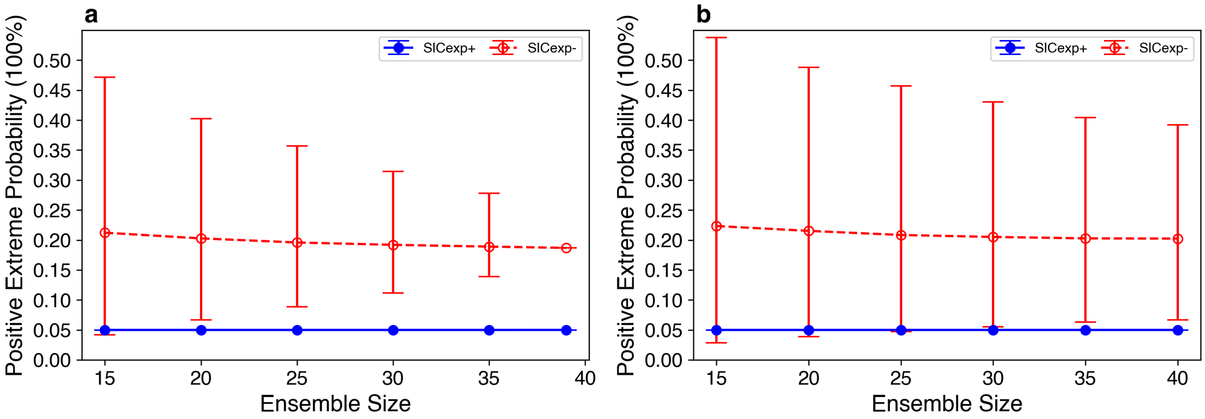

Supplementary Fig. 6. The relationship between the occurrence probability estimates of extreme burning years and ensemble sizes of the CESM-RESFire experiments. a, the probability estimates of extreme burning years based on different sizes of ensemble members in the SICexp- and SICexp+ experiments without replacement in the bootstrap method (see Methods). Note the probabilities of extreme burning years in SICexp+ are always 5% by definition. **b**, as in **a**, but for the probability estimates of extreme burning years with replacement in the bootstrap method. The circles in **a-b** denote the ensemble means of the probability estimates, and the lower and upper whiskers denote the 95% (from 2.5% to 97.5%) inter-percentile ranges of the probability estimates.

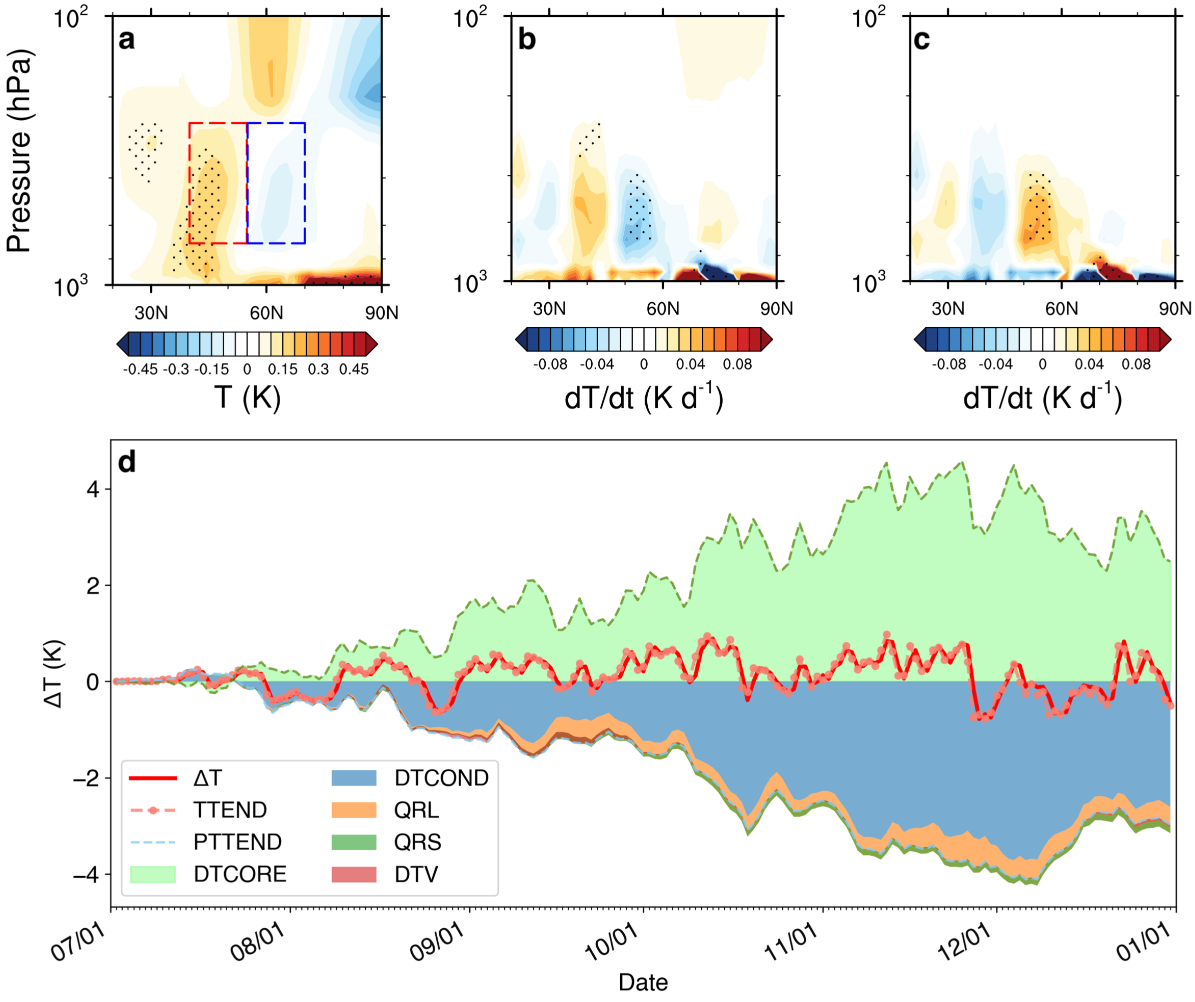

Supplementary Fig. 7. Air temperature and its tendency responses in the CESM-RESFire experiments. **a**, zonally-averaged temperature difference between the SICexp- and SICexp+ sensitivity experiments as shown in Fig. 3c in the main text. The red (blue) box outlines a warm (cool) region in response to the sea-ice perturbation in the SICexp- and SICexp+ experiments, which is averaged for the meridional temperature gradient ($\Delta T = |T_{\text{warm}}| - |T_{\text{cool}}|$) decomposition in **d**. **b**, as in **a**, but for the difference between the SICexp- and SICexp+ experiments in zonally-averaged temperature tendency due to dynamic processes (DTCORE). **c**, as in **a**, but for the temperature tendency difference due to total physical processes (PTTEND). **d**, time series of the meridional gradient in temperature anomaly (ΔT) and its decomposition into

each contributing component, including TTEND as the time integral of the total temperature tendency, PTTEND as the time integral of the temperature tendency due to total physical processes, DTCORE as the time integral of the temperature tendency due to dynamic processes, DTCOND as the time integral of the temperature tendency due to moisture processes, QRL as the time integral of longwave heating rate, QRS as the time integral of shortwave heating rate, and DTV as the time integral of the temperature tendency due to vertical diffusion ($\mathrm{TTEND} = \mathrm{DTCORE} + \mathrm{PTTEND} = \mathrm{DTCORE} + \mathrm{DTCOND} + \mathrm{QRL} + \mathrm{QRS} + \mathrm{DTV}$)). All these terms are calculated as the differences between the SIC- and SIC+ experiments. Stipples in **a-c** show regions that are significantly different from 0 at the 0.1 significance level of a two-sided t-test.

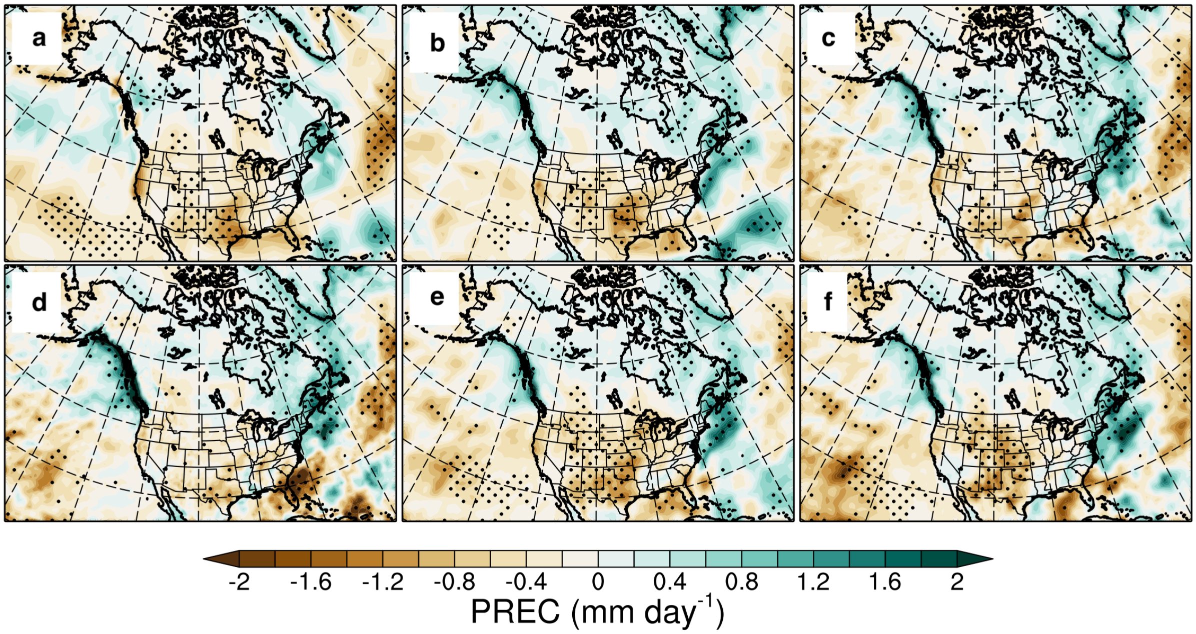

Supplementary Fig. 8. The composite difference of the seasonal average (September to December) total precipitation rates between the SIC- and SIC+ years based on different observational and reanalysis data. a, the precipitation difference based on the GPCP observational data. **b**, as in **a**, but based on the ERA5 reanalysis data. **c**, as in **a**, but based on the MERRA-2 reanalysis data. **d**, as in **a**, but based on the CFSR reanalysis data. **e**, as in **a**, but based on the ERA-interim reanalysis data. **f**, as in **a**, but based on the JRA-55 reanalysis data. Stipples in **a-f** show regions that are significantly different from 0 at the 0.1 significance level of a two-sided t-test.

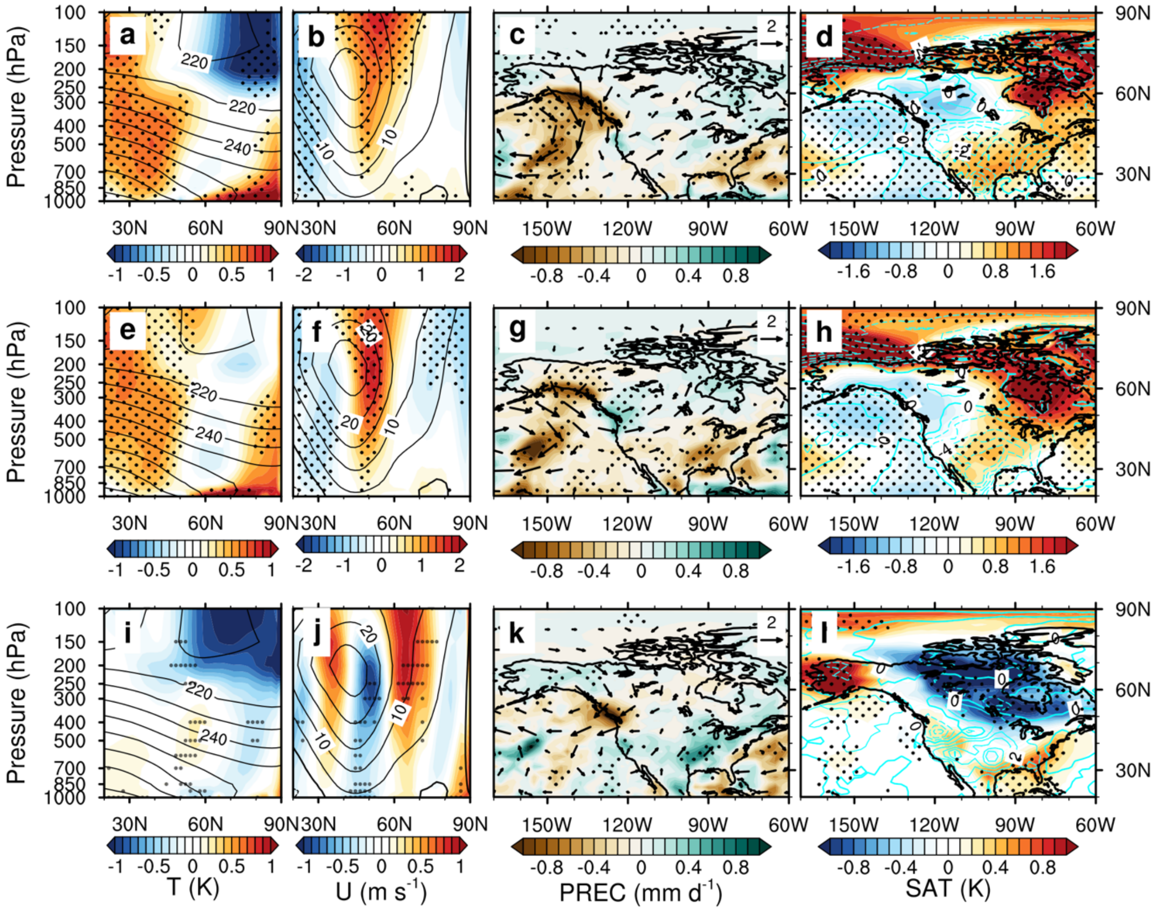

Supplementary Fig. 9. Climate and fire weather changes between the SIC- and SIC+ years based on the CMIP6 amip and amip-piForcing experiments (4-model ensemble). **a**, zonally-averaged (170 °W to 60 °W; as shown in **c**) temperature composite difference (shading) in autumn and early winter (September to December) between the SIC- and SIC+ years (as shown in Fig. 4a) in the amip experiment. The climatology of zonally-averaged temperature in the SIC+ years is also shown (contours; unit: K). **b**, as in **a**, but for zonally-averaged zonal wind in the amip experiment. **c**, as in **b**, but for wind circulation at 500 hPa (arrows; unit: m) and total precipitation rate (shading) differences in the amip experiment. **d**, as in **c**, but for 2-m relative humidity (cyan contours with negative values in dashed lines; unit: %) and 2-m surface temperature (shading) differences in the amip experiment. **e-h**, as in **a-d**, but for the

corresponding variables in the amip-piForcing experiment. **i-l**, as in **a-d**, but for the differences of the corresponding variables between the amip and amip-piForcing experiments. Stipples in **a-l** show regions with all 4 CMIP6 models agreeing on the signs.

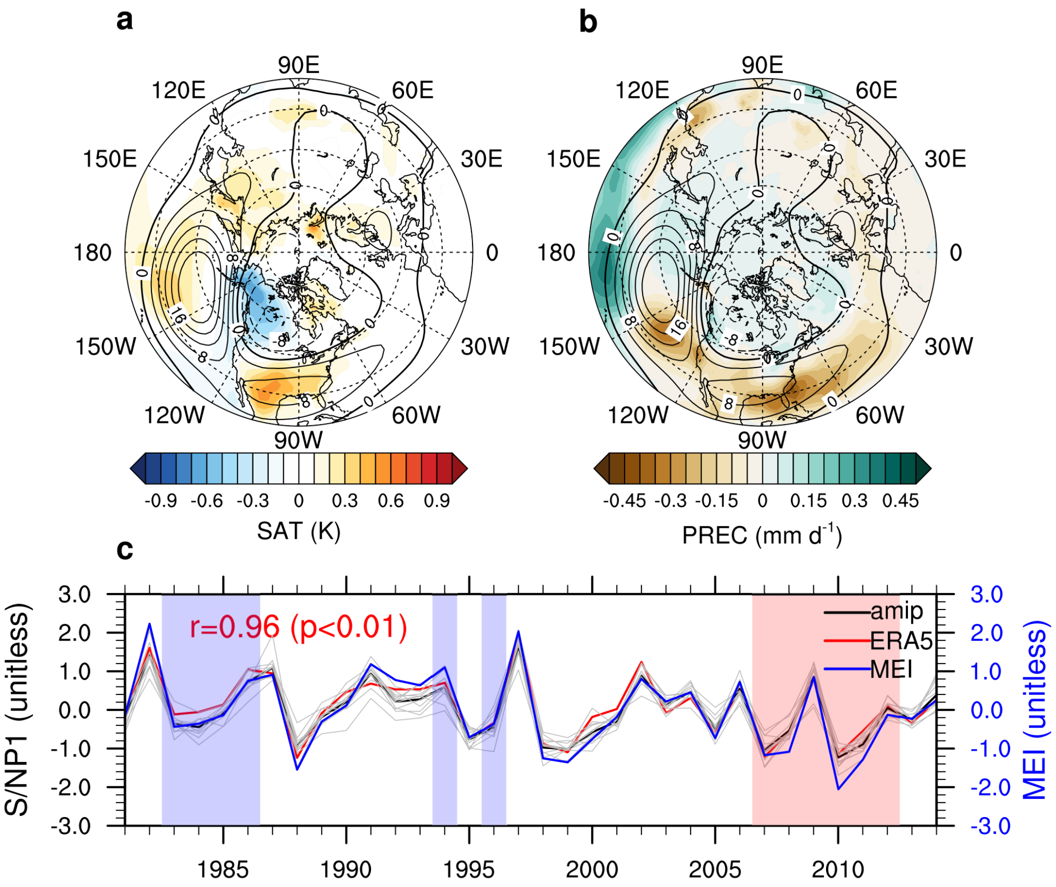

Supplementary Fig. 10. The first group of forced responses (S/NP1) in seasonal mean multi-field weather variables based on the ERA5 and CMIP6 amip experiment ensemble. **a**, the composite difference of S/NP1 forced responses in surface air temperature (shading) and Z500 (contours with negative values in dashed lines; unit: m) of the ERA5 data in autumn and early winter between the SIC- and SIC+ years. **b**, as in **a**, but for total precipitation rates (shading) and Z500 (contours with negative values in dashed lines; unit: m). **c**, timeseries of S/NP1 in the multi-variable meteorological fields based on the ERA5 (red thick line) and amip 12-model ensemble. The black thick line denotes the 12-model ensemble mean and the grey lines denote results from each model. The MEI (blue thick line) and its correlation coefficient with the ERA5 data (red thick line) are also shown for comparison and interpretation. The vertical bar

shading in **c** denotes the SIC- (pink) and SIC+ (blue) years for the composite differences in **a** and **b**.

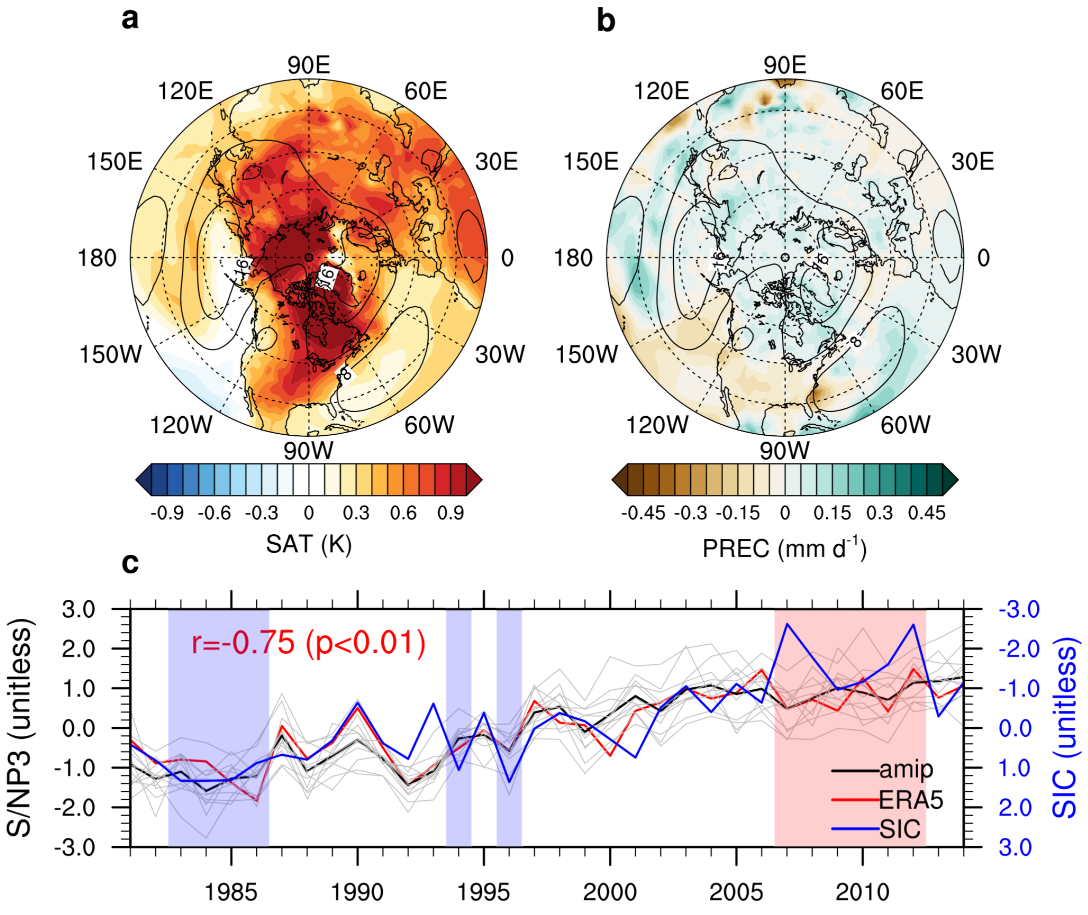

Supplementary Fig. 11. The third group of forced responses (S/NP3) in seasonal mean multi-field weather variables based on the ERA5 and CMIP6 amip experiment ensemble. **a**, the composite difference of S/NP3 forced responses in surface air temperature (shading) and Z500 (contours with negative values in dashed lines; unit: m) of the ERA5 data in autumn and early winter between the SIC- and SIC+ years. **b**, as in **a**, but for total precipitation rates (shading) and Z500 (contours with negative values in dashed lines; unit: m). **c**, timeseries of S/NP3 in the multi-variable meteorological fields based on the ERA5 (red thick line) and amip 12-model ensemble. The black thick line denotes the 12-model ensemble mean and the grey lines denote results from each model. The normalized SIC (blue thick line) and its correlation coefficient with the ERA5 result (red thick line) are also shown for comparison and

interpretation. The vertical bar shading in **c** denotes the SIC- (pink) and SIC+ (blue) years for the composite differences in **a** and **b**.

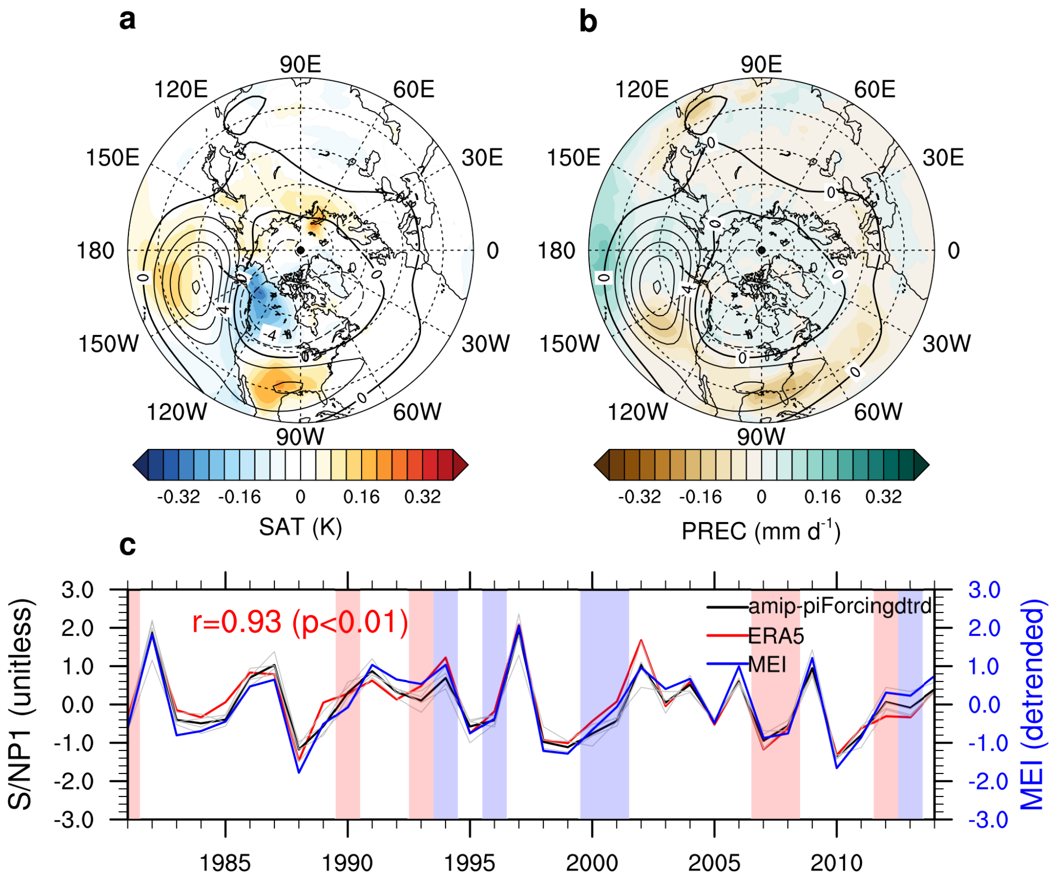

Supplementary Fig. 12. The first group of forced responses (S/NP1) in seasonal mean multi-field weather variables based on the detrended ERA5 and detrended CMIP6 amip-piForcing experiment ensemble. **a**, the composite difference of S/NP1 forced responses in detrended surface air temperature (shading) and detrended Z500 (contours with negative values in dashed lines; unit: m) of the detrended ERA5 data in autumn and early winter between the SICnotrd- and SICnotrd+ years. **b**, as in **a**, but for detrended total precipitation rates (shading) and detrended Z500 (contours with negative values in dashed lines; unit: m). **c**, timeseries of S/NP1 in the multi-variable meteorological fields based on the detrended ERA5 (red thick line) and detrended amip-piForcing 4-model ensemble. The black thick line denotes the 4-model ensemble mean and the grey lines denote results from each model. The detrended MEI (blue thick line) and its correlation coefficient with the ERA5 result (red thick line) are also shown for

comparison and interpretation. The vertical bar shading in **c** denotes the SICnotrd- (pink) and SICnotrd+ (blue) years for the composite differences in **a** and **b**.

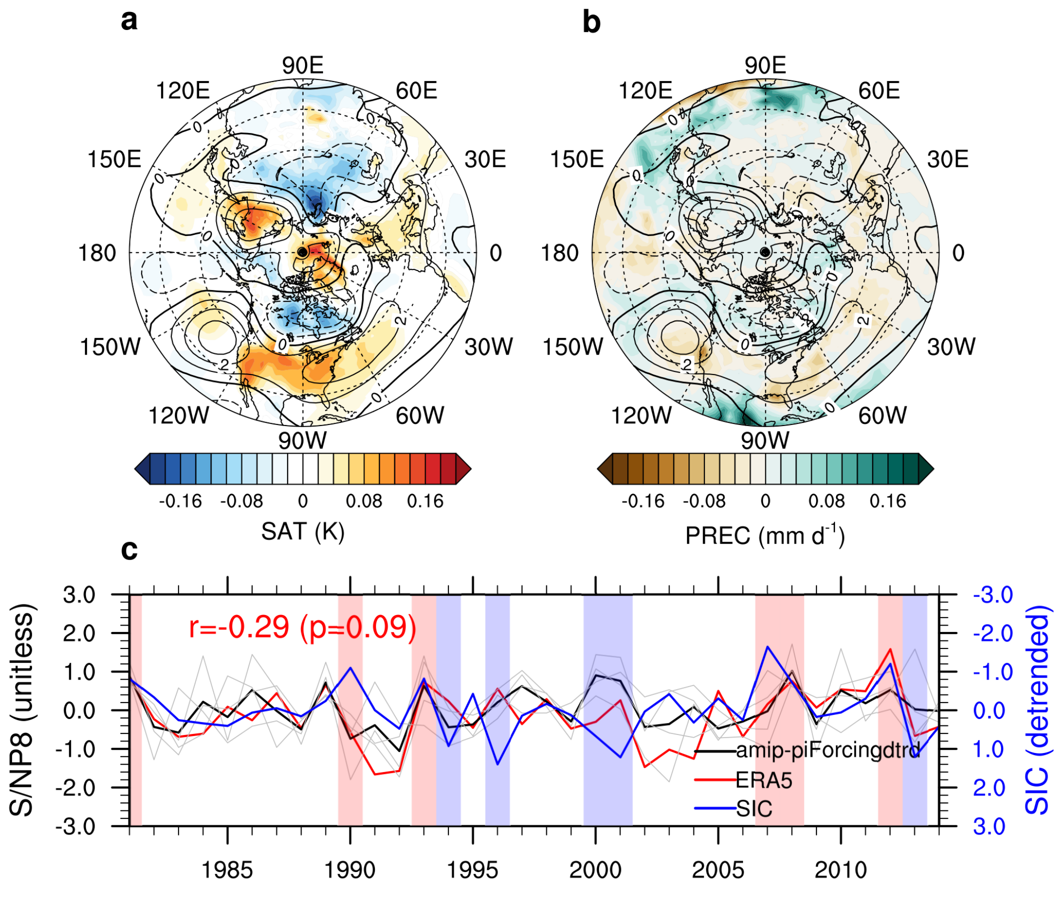

Supplementary Fig. 13. The eighth group of forced responses (S/NP8) in seasonal mean multi-field weather variables based on the detrended ERA5 and detrended CMIP6 amip-piForcing experiment ensemble. **a**, the composite difference of S/NP8 forced responses in detrended surface air temperature (shading) and detrended Z500 (contours with negative values in dashed lines; unit: m) of the detrended ERA5 data in autumn and early winter between the SICnotrd- and SICnotrd+ years. **b**, as in **a**, but for detrended total precipitation rates (shading) and detrended Z500 (contours with negative values in dashed lines; unit: m). **c**, timeseries of S/NP8 in the multi-variable meteorological fields based on the detrended ERA5 (red thick line) and detrended amip-piForcing 4-model ensemble. The black thick line denotes the 4-model ensemble mean and the grey lines denote results from each model. The detrended SIC (blue thick

line) and its correlation coefficient with the ERA5 result (red thick line) are also shown for comparison and interpretation. The vertical bar shading in **c** denotes the SICnotrd- (pink) and SICnotrd+ (blue) years for the composites differences in **a** and **b**.

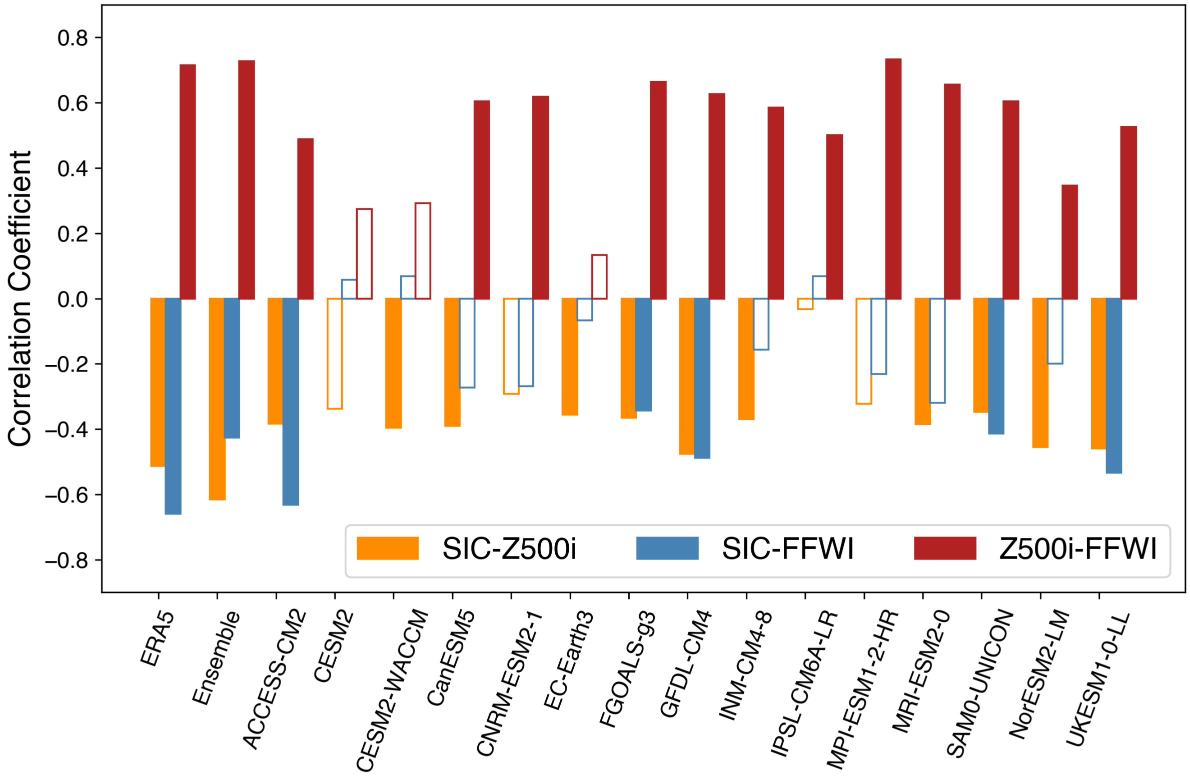

Supplementary Fig. 14. Correlation coefficients among SIC, Z500i, and FFWI in the ERA5 reanalysis data and the amip experiment (1981-2014) based on 15 CMIP6 models and their ensemble mean. The solid-colored bars show correlation coefficients at the 0.05 significance level of a two-sided t-test.

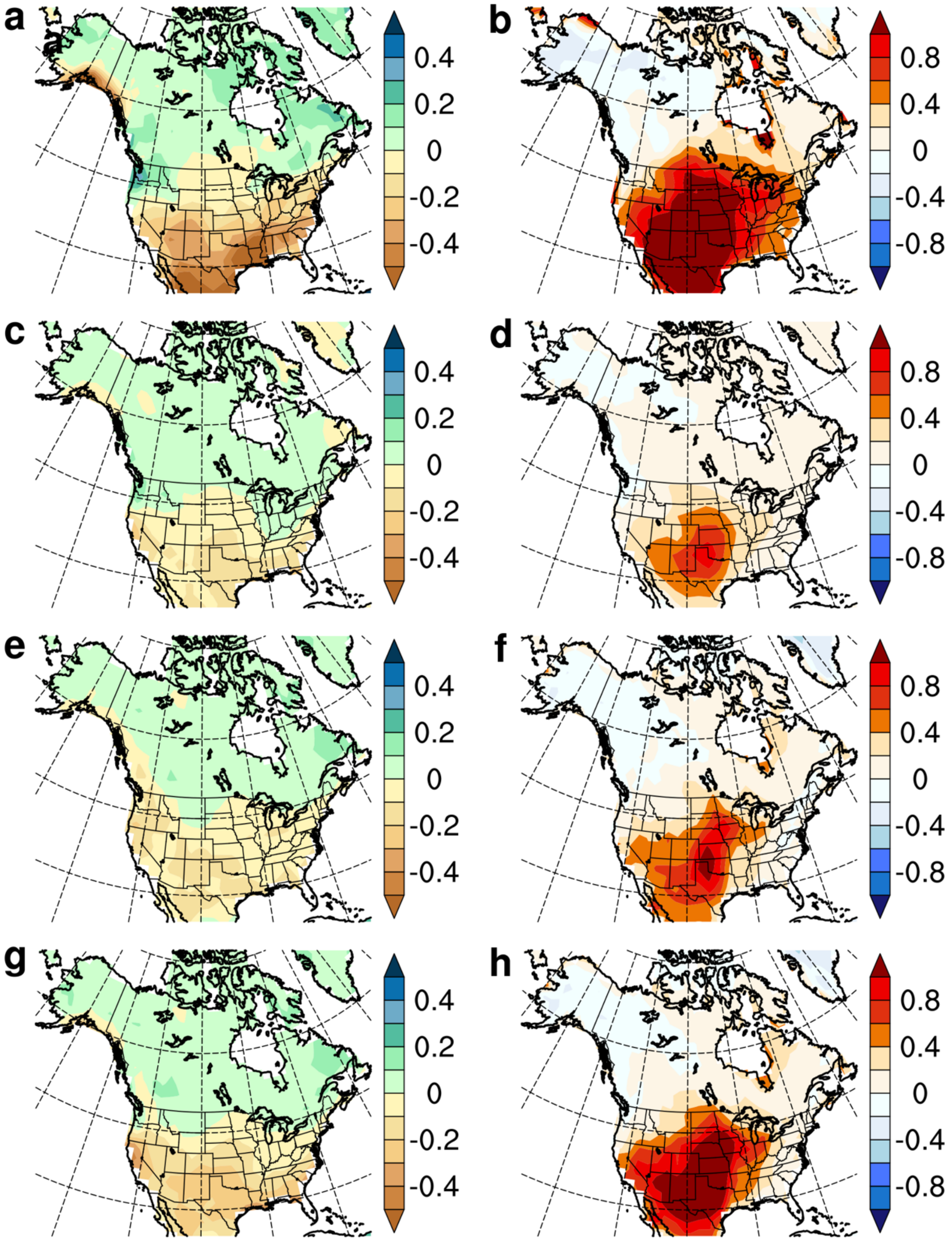

Supplementary Fig. 15. The composite differences of fire weather variables between the SIC- and SIC+ years. **a,** total difference in precipitation (unit: mm d^{-1}) based on the ERA5 data. **b,** total difference in FFWI (unitless) based on the ERA5 data. **c,** differences in precipitation associated with the ENSO-related S/NP1 pattern based on the S/NP analysis using ERA5 and 12

amip model ensemble. **d,** as in **c** but for differences in FFWI. **e,** differences in precipitation associated with the Arctic-related S/NP3 pattern based on the S/NP analysis using ERA5 and 12 amip model ensemble. **f,** as in **e** but for differences in FFWI. **g,** differences in precipitation as the sum of the ENSO-related S/NP1 pattern and the Arctic-related S/NP3 pattern. **h,** as in **g** but for differences in FFWI.

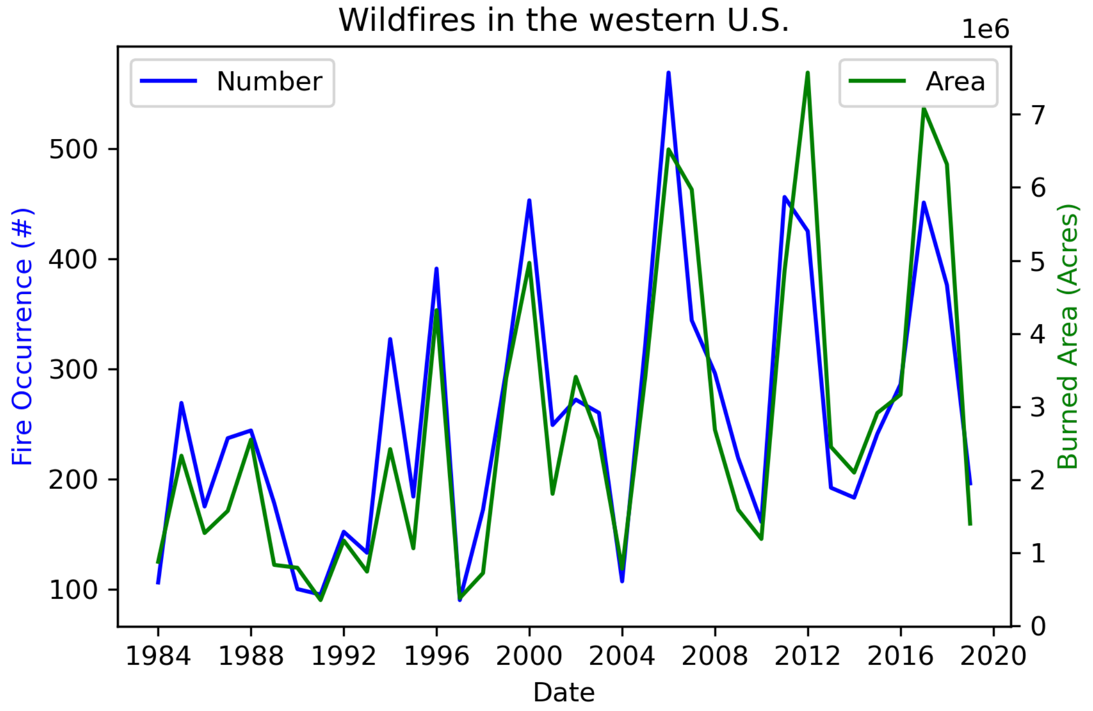

Supplementary Fig. 16. Updated time series of numbers and total burned areas of large wildfires over the western U.S. based on the latest MTBS dataset[7].

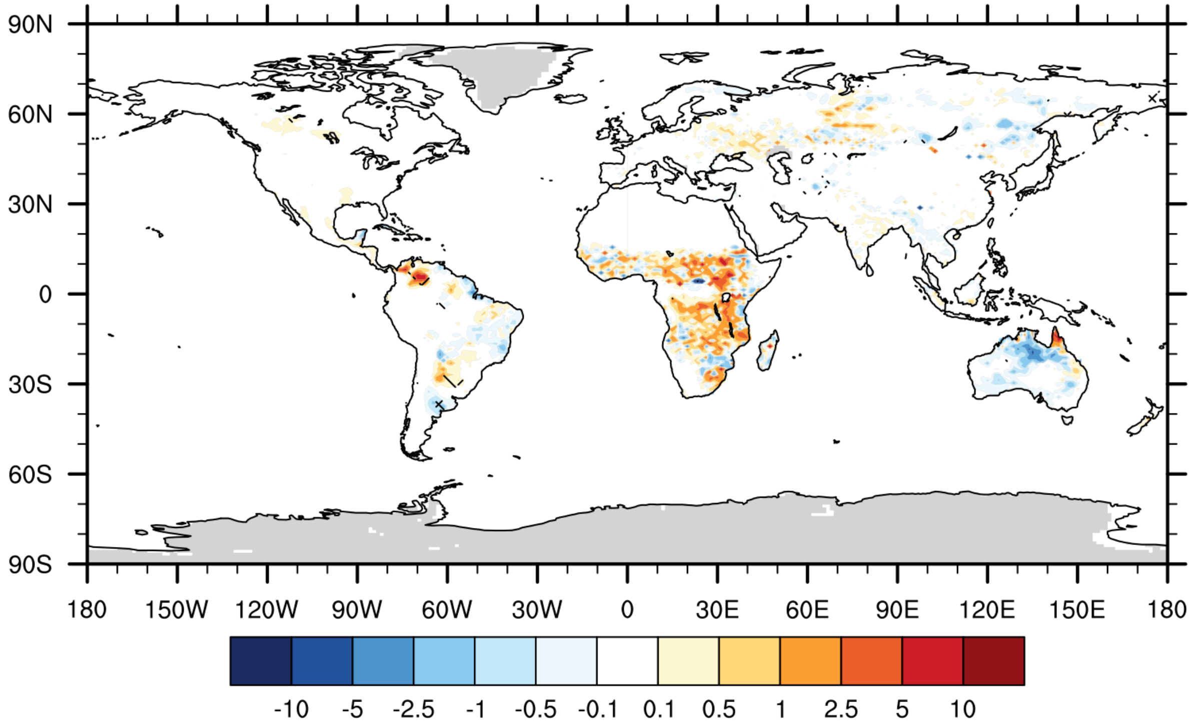

Supplementary Fig. 17. The net fire feedback effect on global burned area (unit: % yr^{-1}) by comparing previous CESM-RESFire sensitivity simulations. The modeling results are based on the difference between control (CTRL1; with full fire feedbacks) and sensitivity (SENS1B; without fire feedback) experiments adapted from Zou et al.[8].

Supplementary Table 1. Correlation coefficients between each pair of fire weather indices based on two different reanalysis datasets and their correlations with the SIC time series

r (*p*-value)	ERA5-based FFWI	ERA5-based FWI	MERRA-2-based FWI
ERA5-based FFWI	1.0	0.81 (<0.001)	0.71 (<0.001)
ERA5-based FWI	0.81 (<0.001)	1.0	0.95 (<0.001)
MERRA-2-based FWI	0.71 (<0.001)	0.95 (<0.001)	1.0
HadISST-based SIC	-0.68 (<0.001)	-0.58 (<0.001)	-0.37 (0.02)

Supplementary Table 2. The modeling settings of the CESM-RESFire sensitivity experiments

Experiment	Control	SICexp+	SICexp-
Time period	40 years	40 years	40 years
Horizontal resolution	1.9°×2.5°	1.9°×2.5°	1.9°×2.5°
Vertical level	70	70	70
Atmosphere	WACCM[a]	WACCM	WACCM
Land	CLM4.5 with RESFire	CLM4.5 with RESFire	CLM4.5 with RESFire
Ocean	Climatology[b]	ICE+ year-average regional SST for the Pacific sector of the Arctic[c]	ICE- year-average regional SST for the Pacific sector of the Arctic[c]
Sea ice	Climatology[b]	ICE+ year-average regional SIC[c]	ICE- year-average regional SIC[c]

[a]: using CAM5 physics package and WACCM_MOZART_MAM3 chemistry package;

[b]: 1981-2010 average based on the HadISST SST and SIC data[9];

[c]: see the main text and Fig. 2a for the region definition;

Supplementary Table 3. The CMIP6 models in the amip experiment used in this study

Model Name	Institute ID	Experiment ID	Ensemble Member
ACCESS-CM2[10]	CSIRO-ARCCSS	amip	r1i1p1f1
CanESM5[11]	CCCma	amip	r1i1p1f1
CESM2[12]	NCAR	amip	r1i1p1f1
CESM2-WACCM[13]	NCAR	amip	r1i1p1f1
CNRM-ESM2-1[14]	CNRM-CERFACS	amip	r1i1p1f2
EC-Earth3[15]	EC-Earth-Consortium	amip	r1i1p1f1
FGOALS-g3[16]	CAS	amip	r1i1p1f1
GFDL-CM4[17]	NOAA-GFDL	amip	r1i1p1f1
INM-CM4-8[18]	INM	amip	r1i1p1f1
IPSL-CM6A-LR[19]	IPSL	amip	r1i1p1f1
MPI-ESM1-2-HR[20]	MPI-M	amip	r1i1p1f1
MRI-ESM2-0[21]	MRI	amip	r1i1p1f1
NorESM2-LM[22]	NCC	amip	r1i1p1f1
SAM0-UNICON[23]	SNU	amip	r1i1p1f1
UKESM1-0-LL[24]	MOHC	amip	r1i1p1f2

Supplementary Table 4. The CMIP6 models in the amip-piForcing experiment used in this study

Model Name	Institute ID	Experiment ID	Ensemble Member
CanESM5[25]	CCCma	amip-piForcing	r1i1p2f1
HadGEM3-GC31-LL[26]	MOHC	amip-piForcing	r1i1p1f3
IPSL-CM6A-LR[27]	IPSL	amip-piForcing	r1i1p1f1
MRI-ESM2-0[28]	MRI	amip-piForcing	r1i1p1f1

278 **Supplementary Table 5. A list of acronyms used in this study**

Category	Acronym	Full Name	Description	First Presence
Geographic zone	WUI	Wildland-Urban Interface	a transition zone between wilderness and land developed by human activity	Introduction
Model type	ESMs	Earth System Models	a coupled modeling system to simulate the interactions of atmosphere, ocean, land, ice, and biosphere and estimate the state of regional and global climate	Introduction
Model name	CESM	Community Earth System Model	an Earth system model maintained by the Climate and Global Dynamics Laboratory (CGD) at the National Center for Atmospheric Research (NCAR)	Introduction
Model name	RESFire	REgion-Specific ecosystem feedback fire model	a recently developed fire model coupled between the land and atmosphere components of CESM	Introduction
Climate variability	AA	Arctic Amplification	the phenomenon that any change in the net radiation balance tends to produce a larger change in temperature near the poles than the planetary average	Introduction
Climate index	SIC	Sea Ice Concentration	the area of sea ice relative to the total at a given point in the ocean	First subsection in Results
Climate index	FFWI	Fosberg Fire Weather Index	a fire weather index to measure the potential influence of weather on a wildfire based on surface air temperature, wind, and relative humidity	First subsection in Results

Agency name	ECMWF	The European Centre for Medium-Range Weather Forecasts	an intergovernmental organisation supported by 34 States providing operational medium and extended-range forecasts and a state-of-the-art super-computing facility for scientific research	First subsection in Results
Climate data	ERA5	the fifth generation ECMWF atmospheric reanalysis of the global climate	the fifth generation ECMWF atmospheric reanalysis of the global climate covering the period from January 1950 to present	First subsection in Results
Composite name	SIC-	minimum SIC years	years with minimum sea-ice concentrations ($<-1\sigma$)during the past four decades	First subsection in Results
Composite name	SIC+	maximum SIC years	years with maximum sea-ice concentrations ($>1\sigma$) during the past four decades	First subsection in Results
Climate variability	ENSO	El Niño-Southern Oscillation	the warm and cool phases of a recurring climate pattern across the tropical Pacific	First subsection in Results
Climate index	SST	sea surface temperature	the water temperature close to the ocean's surface	Second subsection in Results
Experiment name	SICexp-	CESM-RESFire SIC- experiment	CESM-RESFire sensitivity experiment with perturbed Arctic SIC/SST conditions corresponding to the SIC- years	Second subsection in Results
Experiment name	SICexp+	CESM-RESFire SIC+ experiment	CESM-RESFire sensitivity experiment with perturbed Arctic SIC/SST conditions corresponding to the SIC+ years	Second subsection in Results
Climate index	Z500	Geopotential height field at 500 hPa	a vertical coordinate that approximates the actual height of the 500 hPa pressure surface above mean sea-level	Second subsection in Results

Composite name	SICnotrd-	minimum SIC years after detrending	years with minimum sea-ice concentrations (<-1σ) during the past four decades after detrending	Second subsection in Results
Composite name	SICnotrd+	maximum SIC years after detrending	years with maximum sea-ice concentrations (>1σ) during the past four decades after detrending	Second subsection in Results
Climate index	Z500i	Z500 index	Intensity of a fire-favorable circulation pattern in geopotential height field at 500 hPa	Second subsection in Results
Climate forcing agent	GHGs	greenhouse gases	gases that absorb and emit radiant energy within the thermal infrared range, causing the greenhouse effect	Third subsection in Results
Experiment name	amip	the Atmospheric Model Intercomparison Project	An atmosphere only climate simulation using prescribed sea surface temperature and sea ice concentrations but with other conditions as in the Historical simulation	Fourth subsection in Results
Experiment name	amip-piForcing	AMIP SSTs with control forcing	AMIP experiment (with SSTs and Sea Ice the same as in the amip experiment) but with constant pre-industrial forcing levels (anthropogenic & natural)	Fourth subsection in Results
Project name	CMIP6	the Coupled Model Intercomparison Project Phase 6	a collaborative framework designed to improve knowledge of climate change, being the analog of Atmospheric Model Intercomparison Project for global coupled ocean-atmosphere general circulation models	Fourth subsection in Results

Method name	S/NP	the signal-to-noise-maximizing pattern filtering method	a method to identify spatial patterns (linear combinations of empirical orthogonal functions (EOFs)) with the maximum ratio of signal to noise (with signal defined as variance that is agreed upon across an ensemble)	Fourth subsection in Results
Project name	PAMIP	the Polar Amplification Model Intercomparison Project	a coordinated set of numerical model experiments designed to improve our understanding of the Polar amplification phenomenon	Discussion
Project name	FireMIP	the Fire Model Intercomparison Project	a coordinated set of numerical model experiments to assess different fire models to establish the reliability of future projects of changes in fire occurrences and characteristics	Discussion
Climate data	HadISST	the Hadley Centre Sea Ice and Sea Surface Temperature dataset	a combination of monthly globally complete fields of SST and sea ice concentration for 1871-present	Methods
Climate data	MERRA-2	Modern-Era Retrospective Analysis for Research and Applications, Version 2	a NASA atmospheric reanalysis that begins in 1980	Methods
Climate index	FWI	Fire Weather Index	a component of the Canadian Forest Fire Weather Index (FWI) system	Methods
Climate data	GFWED	the Global Fire Weather Database	a fire weather database that integrates different weather factors influencing the likelihood of a vegetation fire starting and spreading	Methods

Project name	GPCP	the Global Precipitation Climatology Project	Datasets provides monthly, pentad, and daily precipitation analysis from surface and satellite measurements for 1979 onwards	Methods
Climate data	CFSR	Climate Forecast System Reanalysis	a third generation global high-resolution reanalysis product coupled atmosphere-ocean-land surface-sea ice system designed to provide the best estimate of the state of these coupled domains over 1979-2017	Methods
Climate data	JRA-55	the Japanese 55-year Reanalysis	the second Japanese global atmospheric reanalysis project covering 55 years since 1958	Methods
Project name	MTBS	the Monitoring Trends in Burned Severity program	an interagency program whose goal is to consistently map the burn serverity and extent of large fires across all lands of the US from 1984 to present	Methods
Climate index	BAC	Fractional burned area change	Fractional changes of the monthly total burned area of large wildfires averaged over the western US between the minimum and maximum SIC years	Methods
Model name	CLM4.5	the Community Land Model version 4.5	the land model for the Community Earth System Model (CESM)	Methods
Model name	WACCM	the Whole Atmosphere Community Climate Model	a comprehensive numerical model spanning the range of altitude from the Earth's surface to the thermosphere in the Community Earth System Model (CESM)	Methods

Experiment name	CTRL	CESM-RESFire control run	the CESM-RESFire control simulation constrained by climatological surface boundary conditions in order to provide intial conditions for the other two sensitivity experiments	Methods
Climate index	NFDRS	the National Fire Danger Rating System	a system that allows fire managers to estimate today's or tomorrow's fire danger for a given area	Methods
Climate index	BI	burning index	the burning index in NFDRS	Methods
Climate index	MEI	the Multivariate El Niño-Southern Oscillation index	the time series of the leading combined Empirical Orthogonal Function (EOF) of five different variables (sea level pressure (SLP), sea surface temperature (SST), zonal and meridional components of the surface wind, and outgoing longwave radiation (OLR)) over the tropical Pacific basin (30°S-30°N and 100°E-70°W)	Methods
Method name	EOF	Empirical Orthogonal Function	a multivariate statistical technique partitioning a field into mathematically orthogonal (independent) modes	Methods
Model variable	TTEND	total temperature tendency	total temperature tendency	Methods
Model variable	DTCORE	T tendency due to dynamical core	temperature tendency driven by dynamic processes	Methods
Model variable	PTTEND	T tendency due to total physics	temperature tendency driven by physical processes	Methods
Model variable	DTCOND	T tendency due to precipitation physics	temperature tendency driven by moisture processes	Methods
Model variable	QRL	Longwave heating rate	temperature tendency driven by longwave heating	Methods
Model variable	QRS	Solar heating rate	temperature tendency driven by shortwave heating	Methods

Model variable	DTV	T tendency due to vertical diffusion	temperature tendency driven by vertical diffusion	Methods
Model variable	TTGW	T tendency due to gravity wave	temperature tendency driven by gravity wave drag	Methods

Supplementary References:

1 Fosberg, M. A. Weather in wildland fire management: the fire weather index, in *Conference on Sierra Nevada Meteorology.* 1-4 (1978).

2 Van Wagner, C. E. Development and Structure of the Canadian Forest Fire Weather Index System. Canadian Forest Service, Ottawa, Canada (1987).

3 Vitolo, C. et al. ERA5-based global meteorological wildfire danger maps. *Sci Data* **7**, 216, https://doi.org/10.1038/s41597-020-0554-z (2020).

4 Field, R. D. *et al.* Development of a Global Fire Weather Database. *Nat Hazard Earth Sys* **15**, 1407-1423 (2015).

5 Copernicus Climate Change Service (C3S). ERA5: Fifth generation of ECMWF atmospheric reanalyses of the global climate . Copernicus Climate Change Service Climate Data Store (CDS). https://cds.climate.copernicus.eu/cdsapp#!/home (2017).

6 Rienecker, M. M. *et al.* MERRA: NASA's Modern-Era Retrospective Analysis for Research and Applications. *J Climate* **24**, 3624-3648 (2011).

7 Eidenshink, J. C., Schwind, B., Brewer, K., Zhu, Z.-L., Quayle, B., and Howard, S. A Project for monitoring trends in burn severity. *Fire Ecology Special Issue* **3**, 19 (2007).

8 Zou, Y. F. *et al.* Using CESM-RESFire to understand climate-fire-ecosystem interactions and the implications for decadal climate variability. *Atmos Chem Phys* **20**, 995-1020 (2020).

9 Rayner, N. A. *et al.* Global analyses of sea surface temperature, sea ice, and night marine air temperature since the late nineteenth century. *J Geophys Res-Atmos* **108** (2003).

10 Dix, M. *et al.* (Earth System Grid Federation, 2019).

11 Swart, N. C. *et al.* (Earth System Grid Federation, 2019).

12 Danabasoglu, G. (Earth System Grid Federation, 2019).

13 Danabasoglu, G. (Earth System Grid Federation, 2019).

14 Seferian, R. (Earth System Grid Federation, 2018).

15 Consortium, E. C.-E. (Earth System Grid Federation, 2019).

16 Li, L. (Earth System Grid Federation, 2019).

17 Guo, H. *et al.* (Earth System Grid Federation, 2018).

18 Volodin, E. *et al.* (Earth System Grid Federation, 2019).

19 Boucher, O., Denvil, S., Caubel, A. & Foujols, M. A. (Earth System Grid Federation, 2018).

20 Jungclaus, J. *et al.* (Earth System Grid Federation, 2019).

21 Yukimoto, S. *et al.* (Earth System Grid Federation, 2019).

22 Seland, Ø. *et al.* (Earth System Grid Federation, 2019).

23 Park, S. & Shin, J. (Earth System Grid Federation, 2019).

24 Tang, Y. *et al.* (Earth System Grid Federation, 2019).

25 Cole, J. N. S. *et al.* (Earth System Grid Federation, 2019).

26 Webb, M. (Earth System Grid Federation, 2019).

27 Boucher, O., Denvil, S., Caubel, A. & Foujols, M. A. (Earth System Grid Federation, 2019).

28 Yukimoto, S. *et al.* (Earth System Grid Federation, 2019).
